# Reflections on and recommendations from the OCEANIC study CYP and parent advisory group: a Patient and Public Involvement and Engagement case study

**DOI:** 10.1186/s40900-026-00904-6

**Published:** 2026-05-19

**Authors:** Emma Popejoy, Julie Menzies, Aysha Sheikhi, Sally O’Loughlin, Fred O’Loughlin, Deborah Barker, Rachael White, Jos M. Latour, Elizabeth S. Draper, Philip Quinlan, Jane Coad, Joseph C. Manning

**Affiliations:** 1 Nottingham Children’s Hospital, Nottingham University Hospitals NHS Trust, Nottingham, Nottinghamshire, UK; 2 School of Healthcare, University of Leicester, Leicester, UK; 3 School of Health Sciences, University of Nottingham, Nottingham, Nottinghamshire, UK; 4 Paediatric Intensive Care, University Hospitals Bristol and Weston NHS Foundation Trust, Bristol, UK; 5 University of the West of England, Bristol, UK; 6 OCEANIC Study Children, Young People’s and Parent Advisory Group Member, Nottingham, UK; 7 School of Nursing and Midwifery, University of Plymouth, Plymouth, UK; 8 Nursing Department, Zhongshan Hospital of Fudan University, Shanghai, China; 9 Department of Population Health Sciences, University of Leicester, Leicester, Leicestershire, UK; 10 Faculty of Medicine & Health Sciences, University of Nottingham, Nottingham, Nottinghamshire, UK; 11 University Hospital Coventry and Warwickshire NHS Foundation Trust, Coventry, UK

**Keywords:** Patient and Public Involvement Engagement, Paediatric critical care, Paediatric intensive care, Children and young people, Longitudinal research, PPIE case study

## Abstract

**Background:**

Patient and Public Involvement and Engagement (PPIE) is increasingly recognised as essential to the relevance, feasibility, and impact of paediatric intensive/critical care research. However, reporting of PPIE processes and outcomes, particularly in child health studies, remains limited, often lacking detail on sustained involvement and its influence across the research lifecycle.

**Case study:**

This case study presents the PPIE approach embedded within the OCEANIC Study, a multi-centre longitudinal mixed-methods investigation into the outcomes and support needs of children and families following paediatric intensive care unit (PICU) discharge. The study uniquely sustained engagement with a Children, Young People and Parent Advisory Group (CYPPAG) over eight years, aligning with the National Institute for Health and Care Research (NIHR) standards for public involvement. We detail the strategies used to foster inclusive, respectful, and impactful collaboration, including co-development of study materials, iterative feedback on recruitment and retention strategies, and involvement in qualitative data interpretation and dissemination. The advisory group’s contributions shaped study design, enhanced recruitment, and informed dissemination, including co-authorship and conference presentations. PPIE impact was explored qualitatively through CYPPAG feedback, tracking of study changes informed by the CYPPAG, and ongoing reflection within the research team.

**Conclusion:**

The OCEANIC Study demonstrates the feasibility and value of longitudinal, embedded PPIE in paediatric critical care research. It offers a novel model for sustained advisory group involvement, evidencing how meaningful collaboration can improve research relevance, rigour, and reach. This case study provides actionable insights for researchers seeking to integrate PPIE across the research continuum and highlights the therapeutic and empowering impact of involvement for contributors.

## Abstract (plain language summary)

Patient and public involvement and engagement (PPIE) means working together with patients, families, and the public to make research more useful. PPIE is important but can be hard to do well, especially in children’s intensive care research. This article explains how we involved children, young people, and parents in the OCEANIC Study, which looked at what happens to families after a child leaves intensive care. The advisory group felt respected and listened to, thanks to clear communication and regular updates. Their ideas helped shape the study and how we shared the results. They had a good experience being involved. This article gives advice for others doing research with children and families.

## Background

### Patient and Public Involvement and Engagement in paediatric research

Patient and Public Involvement and Engagement (PPIE) encompasses initiatives to include patients, family members, carers and members of the public in developing and improving health services and research. The principle underlying PPIE is that research is carried out ‘with’ or ‘by’ members of the public, rather than ‘to’, ‘about’ or ‘for’ them [1]. The active involvement of patients and the public in the design and delivery of health research has been increasingly encouraged, with benefits cited as enhanced relevance of the research, improved acceptability of data collection methods, and improved recruitment and retention, alongside enhanced research impact and public empowerment [2]. Key to successful PPIE is an active partnership between patients, carers, members of the public and researchers to influence and shape research [3].

The National Institute for Health and Care Research (NIHR) standards for Patient and Public Involvement [4] provide six key elements to define what good public involvement in research should look like:

- Public involvement opportunities should be accessible and reflect diversity of public experience and insight, so that it leads to treatments and services which reflect these needs.
- Researchers need to work together with patient and public collaborators in a way that values all contributions, and that builds and sustains mutually respectful and productive relationships.
- Collaborators should be provided with support and learning opportunities that build confidence and skills for involvement in research.
- Researchers need to communicate clearly using plain language for well-timed and relevant communications.
- Patient and public collaborators should be involved in research management, regulation, leadership and decision-making to help with transparency and public trust.
- The last principle reflects the importance of articulating impact; understanding the changes, benefits and learning gained from the insights and experiences of patients, carers and the public.

There is a growing commitment among the research community to ensuring that PPIE is undertaken and that its impact is disseminated. However, recent reviews have reported that studies often fail to report the approach to, or process for, involvement [5], or the impacts of PPIE activities [6]. This challenge is particularly evident for paediatric intensive/critical care research, with reviews highlighting only a handful of papers reporting on PPIE in this setting [7, 8]. In the paediatric intensive/critical care setting, there is limited evidence reporting examples of sustained PPIE throughout the research lifecycle, rather it is more frequently discussed as occurring within one or two specific phases of the research. Conducting PPIE using this approach not only requires significant time to set up and facilitate [9] but also fails to consider the contribution possible within all stages of the research cycle [3]. Even within research reports funded by the NIHR, which provides reporting guidance for PPIE, there is variation in how it is reported, as described in a review of NIHR funded child health research [10]. Only four out of thirty-two child health reports featured detail about the involvement of Children and Young People (CYP), with a lack of clarity about who was involved, why, and what outcomes and impact involvement had on the research process, on CYP and/or on researchers [10].

### Paediatric intensive/critical care context

Much of the clinical practice within paediatric intensive/critical care is not supported by high-quality evidence and practice surrounding even commonly used interventions can vary widely between units and individual intensivists [12]. Conducting clinical trials regarding the care and management of critically ill children is therefore a priority, but difficult to achieve [13]. There are important considerations around patient eligibility, consent models, recruitment strategies, trial interventions and randomisation, as well as consideration of outcome measures [14, 15]. With recognition that research is a vital component of a high-quality service [16], there is an aspiration that research should be the standard of care for all critically ill children and their families [17]. PPIE is therefore vital to demonstrate to funders and research ethics committees that the research question is important and that researchers have worked to address the feasibility and acceptability of the trial design and information materials [18].

It is clear that providing detailed information about the conduct and impact of PPIE in paediatric intensive/critical care research is an area in need of improvement, including detailing the impact of PPIE on the people involved, research process and PPIE processes, with specific reference to virtual PPIE activity [11]. Reporting the processes and outcomes of CYP involvement more rigorously may help child health researchers involve CYP more meaningfully by learning from the experiences, enablers and challenges faced by other researchers [10].

This case study will report on the PPIE conducted within a study investigating the Outcomes of ChildrEn and fAmilies iN the first year after paediatric Intensive Care (The OCEANIC Study). It will describe and reflect on the processes, impacts and lessons learned from sustained advisory group involvement. CYPPAG member co-authors were invited, as part of the manuscript drafting process, to reflect on their experiences of involvement in the OCEANIC Study and provided written comments and quotations, which are included in this paper to illustrate their perspectives on the PPIE process.

The OCEANIC Study provides a useful example for understanding effective PPIE practices by demonstrating how meaningful and sustained involvement can be achieved within a longitudinal study, in an emotive context, and with families who have significant competing priorities. While the context is specific, the underlying approaches to relationship-building, flexibility and collaboration are transferable to similarly challenging research contexts. Therefore, this case study offers practical, transferable learning for researchers aiming to embed PPIE effectively in their own studies.

## Case study: the OCEANIC study

**Fig. 1:**
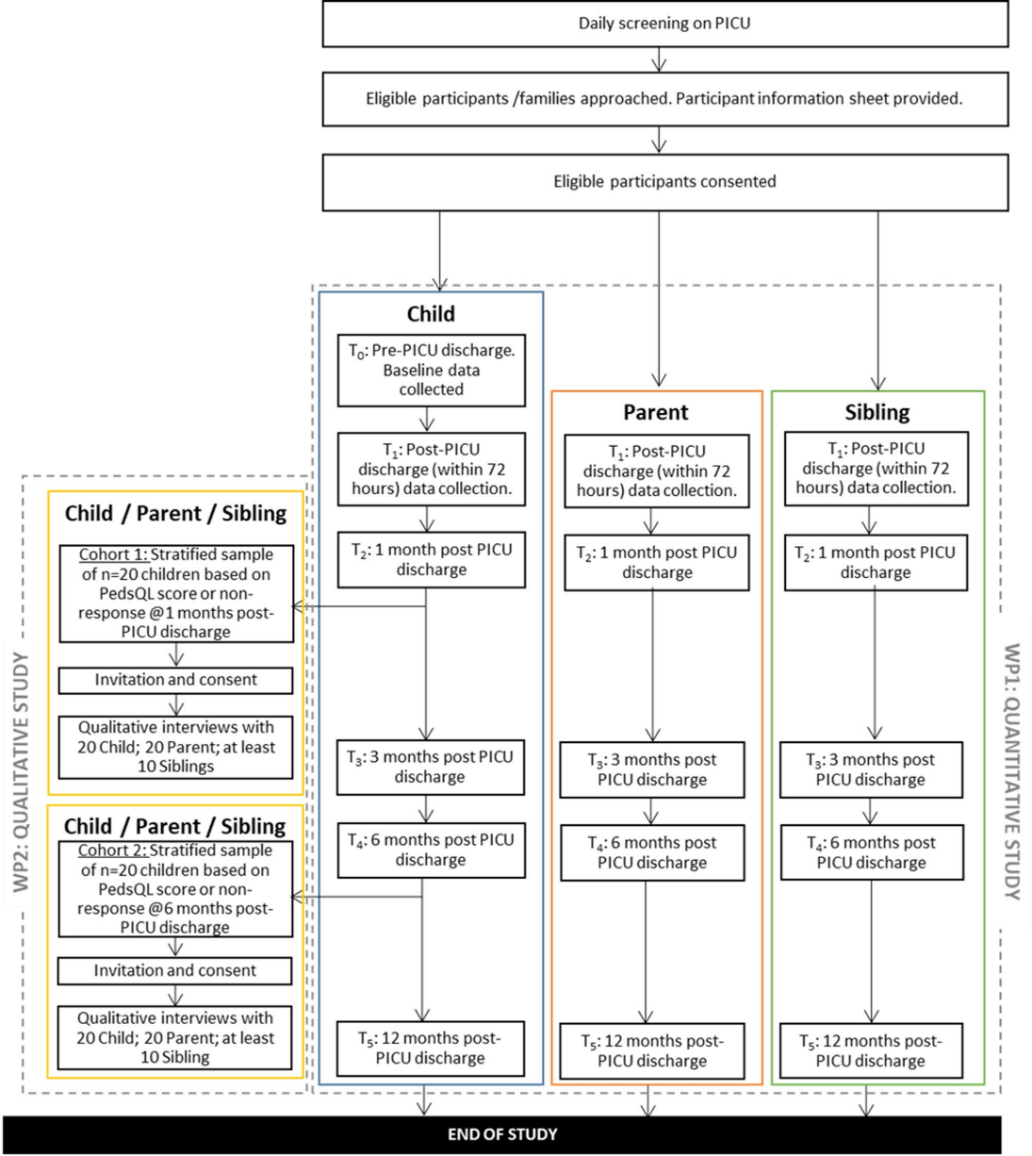
OCEANIC study overview

The OCEANIC Study is a multi-centre longitudinal mixed methods study. The study was conducted across ten PICU’s in England. It was a mixed-methods study involving two linked work-packages (Fig. 1). It aimed to identify the longitudinal physical-, cognitive-, emotional- and social-health outcomes and the support needs of children and their families in the first year following a PICU discharge. The study procedures are described in detail elsewhere [19], but a brief overview is provided here to contextualise the PPIE activity.

### Overview of study methodology

Work-package 1 (WP1) involved: (i) the collection of routinely collected PICU activity data which was downloaded from the Paediatric Intensive Care Audit Network (PICANet) database; and (ii) completion of a range of measures by the child (or parent as proxy), their parent, and sibling (if appropriate) in the 12 months following their PICU discharge. Data collection measures were selected to cover the four domains highlighted within the Post-Intensive Care Syndrome in Pediatrics (PICS-p) framework [20] which were physical, cognitive, emotional and social health.

Prior to the child’s PICU discharge, their baseline status was collected through completion of questionnaires by the parent/child (if able) and follow up data collection consisted of the completion of questionnaires at 1-, 3-, 6- and 12-months post-PICU discharge. Parent and sibling data was collected at the same time points, except for baseline, which was not collected for those participant types. Following hospital discharge, questionnaire data were collected primarily online (through the cloud-based REDCap system), on paper copies, or via telephone (by a research nurse or the central study team), depending on family preference.

Work-package 2 (WP2) consisted of semi-structured interviews with forty families. Two cohorts of families were interviewed: twenty families at 1–3 months post-PICU discharge and a separate cohort of twenty families at 6–9 months post-PICU discharge. Interviews were conducted via online video conferencing or telephone, according to participant preference. Families were stratified based on PedsQL Score, geographical location, PICU presenting condition, age and ethnicity. Interviews focused on the care and support needs of the child and family following the PICU discharge. Interviews were transcribed verbatim and a modified five stage Framework Analysis approach used to analyse the data.

### Patient and Public Involvement and Engagement within the OCEANIC study

The OCEANIC Study research team were committed to ensuring that there was ongoing and meaningful PPIE through the life course of the study. Numerous PPIE and stakeholder activities were undertaken prior to funding and commencement of the OCEANIC Study, as outlined in the protocol [19]. The initial PPIE undertaken included a diverse set of voices including parent/carers, CYP PICU survivors and siblings, and highlighted the need for longitudinal research focusing on optimising long-term outcomes and the identification of unmet needs, as well as providing advice regarding study methods and terminology.

### Application of the NIHR standards for Patient and Public Involvement

The PPIE conducted within the OCEANIC Study will be addressed in relation to the six NIHR standards for Patient and Public Involvement [4]. This case study is reported in accordance with the Guidance for Reporting Involvement of Patients and the Public (GRIPP2) [21].

#### Accessibility and reflecting diversity

Upon receipt of funding, the OCEANIC Children, Young People’s and Parent Advisory Group (CYPPAG) was established, with the aim of providing advice on the management of the research, developing participant information resources, contributing to the study report, and dissemination of research findings. A diverse group of members, reflecting the study eligibility criteria [19], were sought to ensure that a breadth of lived experience was represented. To maximise diversity, families were purposively approached on one PICU based on presenting condition and ethnicity, with additional geographical diversity achieved through existing contacts. Over the course of the study, a total of seven CYPPAG members were involved: one young person, one sibling and five parents of CYP who had had a PICU admission (Table 1). Four members were recruited by JM through her role as a nurse researcher, who sensitively approached families known to the unit either during admission or following step-down to the ward, at appropriate time points. One member contacted JCM directly to express interest in advising on the study, and one member was recruited through a personal, non-clinical connection to EP. The group membership changed over the course of the study, as other commitments took priority, or additional interested individuals were identified and recruited.

The CYP member who had had a previous PICU admission was in her mid-teens when she joined the OCEANIC Study CYPPAG, alongside her younger sister. Both CYP decided to withdraw their involvement in the study, after approximately three years, when the PICU survivor commenced university and wanted to ‘move on’ from that chapter of her life.

**Table 1:** Characteristics of the OCEANIC CYPPAG membership

Characteristic	Number
Participant Type	
Mother Father CYP PICU Survivor CYP Sibling	4 1 1 1
Ethnicity	
White British Arab	6 1
Time since PICU admission at enrolment	
<3 years >20 years	6 1
PICU Presenting condition*	
Cardiac Metabolic Guillan Barre Syndrome Other	2 1 1 1
Region	
West Midlands North-West South-East	5 1 1

*Presenting condition of the 5 related CYP (two CYP were represented by two CYPPAG members)

The group were diverse in their experiences and accessed different PICU’s. This diversity of background, treating centre and underlying condition helped to ensure that a variety of perspectives were represented, which may have impacted on study recruitment and retention. The study website included a section which housed statements from CYPPAG members about why they believed the study was important; one parent also translated her statement into Arabic. We recognise that whilst we achieved some degree of diversity in the group membership, this was not fully representative of the study population and presented a limitation of the advice and guidance that the CYPPAG provided.

Through reflective discussions at CYPPAG meetings during the OCEANIC Study and sustained contact in relation to other projects since, CYPPAG members stated that their experience of advising on the study was positive. This was facilitated through frequent contact between researchers and CYPPAG members, flexibility in preferred methods of communication, attention to accessibility requirements for face‑to‑face meetings, and the provision of edible incentives for online meetings. The initial face‑to‑face meeting was held centrally in the Midlands. This location was chosen to support attendance, with members’ travel expenses covered and time remunerated. The venue was selected for its accessibility to accommodate mobility requirements, and dietary needs and modes of nutrition were sensitively discussed with participants in advance. Where additional care needs were identified, CYPPAG members were supported to attend with a carer (required for one member). For convenience and due to the COVID context, the majority of meetings were conducted virtually.

#### Building respectful and productive relationships

The OCEANIC Study CYPPAG has maintained channels of communication and ensured lasting engagement over a period of 8 years, from their recruitment in 2018, to present. This is testament to the establishment of respectful and productive relationships from the outset. Key to building these relationships was a deep-seated commitment from the research team to understanding the lived experiences of CYPPAG members, and an awareness of the necessity to incorporate these insights into the study materials, design, analysis and dissemination. One of the CYPPAG members commented:

*I feel that your respect for our lived experience and your warmth and openness created an ethos that underpinned the work and was critical to our continued involvement. I know from conversations with the other participants that we all felt heard and valued and believe that this aspect should be highlighted more strongly as it was critical to our continued engagement. I have been involved in several advisory groups in different settings over the years and found in this project a willingness to listen and to learn on the part of the team that I found deeply moving and exceptional and kept me engaged and feeling that the time and energy that I was putting in was worthwhile. I think it also played a large part in our willingness to take time out of our busy schedules to travel to Edinburgh and share our experiences [at the conference].* (DB)

Whilst it is not possible to create a roadmap for establishing these relationships, emotional intelligence and a commitment to active listening on the part of the research team is essential and will create the foundations required.

In order to establish productive relationships within the CYPPAG, members highlighted the importance of having the ‘right size’ group, which they identified that OCEANIC Study CYPPAG provided. They noted that having between 4 and 6 PPIE advisors present provided the best opportunity to have their voice heard but not feel too great a weight of responsibility.

Refreshments were provided to CYPPAG members to enjoy during the virtual meetings. Afternoon tea boxes were sent to members in advance of the meeting and signalled the study team’s appreciation of their time, which were well received:

*Even though COVID disrupted a few things*,* the meetings continued*,* and I felt like I was heard. With the personal touches of sending gift Lunch boxes that I looked forward to receiving with every meeting. This helped me feel appreciated.* (AS).

In addition, CYPPAG members were remunerated for their time in line with NIHR guidance, further recognising the value of their contributions and supporting respectful and productive relationships.

The disruption caused by the COVID-19 pandemic did not appear to impact CYPPAG Members’ commitment to the study or their engagement with meetings. Despite the pandemic occurring almost immediately after the first CYPPAG meeting, their continued engagement in the study resulted from both members’ belief in the importance of the study, and the ethos of collaboration which they experienced during the first face-to-face CYPPAG meeting.

#### Learning opportunities for PPIE advisors

The OCEANIC Study researchers were keen that the CYPPAG members had access to learning and development opportunities during their involvement. At the first in-person CYPPAG meeting, a PowerPoint presentation guided members to understand the purpose and approach to PPIE through discussion of the NIHR standards [4], the anticipated time commitments and were involved in discussions about how they would best feel able to contribute. The discussion focused on clarifying the role of PPIE advisors, reassuring members that the expectation was simply to share their views on the study, and related issues, in an open and supportive environment. It was apparent that they were largely well‑educated and confident individuals, who did not require additional training or support to facilitate their participation in CYPPAG meetings; however, this assessment was based on early engagement rather than a formal discussion of perceived training needs. Reflecting on this, we would recommend explicitly addressing training and support requirements at initial engagement, an approach the research team will adopt in future PPIE endeavours.

Towards the end of the study however, the research team recognised the imbalance in opportunities for learning and were keen to ensure that CYPPAG members were offered development and educational opportunities. Subsequently a webinar was organised to provide CYPPAG members with an overview of the research regarding the psychological impact following PICU-discharge and the services available nationally. This was evaluated well by members and made them feel valued. Their attendance at a conference to present their PPIE involvement also provided an opportunity to learn about clinical and research advancements in PICU.

#### Timely and clear communication

During the life-course of the study the CYPPAG met with the Chief Investigator (JCM) and Research Fellow (EP) twice yearly. The first OCEANIC CYPPAG was conducted face to face in February 2020, three months after the study first opened to participant recruitment. The meeting was attended by the Chief Investigator and Research Fellow, along with three parent members and the PICU Survivor. PICU Research Nurses from one of the study sites also attended to provide an overview of recruitment challenges. Almost immediately following the first face to face meeting, the Covid-19 pandemic resulted in a five month pause to the study (March-August 2020) and then a second, shorter, pause in February-March 2021. CYPPAG meetings were suspended during the pauses and subsequently transitioned to an online format, resuming their twice-yearly frequency from September 2021. Meetings followed a consistent format, beginning with a PowerPoint presentation at the outset to re-orientate members to the study and provide a reminder of the study aims and methods. Meetings then covered specific research challenges or data analysis, depending on the stage of the research, and members were invited to provide any comments, suggestions or feedback on these. The study team felt that it was important to re-orientate the CYPPAG to the study each time, with a reminder of the aims, objectives and methods of the study, as they were aware of the wider commitments of the members, which included busy family lives, ongoing health concerns/check-ups/appointments and their paid employment, to name a few.

Meeting minutes were compiled and summaries sent to CYPPAG members following each meeting, including whether and how their advice had been incorporated into the study processes. A WhatsApp group was also formed with members upon their request, to help make communication regarding the study more accessible.

#### Involvement in research management and decision making

PPIE was central to decision making within the OCEANIC Study. Prior to application for funding and prior to convening the CYPPAG, PPIE advice informed selection of the outcome measures for the study, methods of data collection and development of the Participant Information Sheets.

**Fig. 2:**
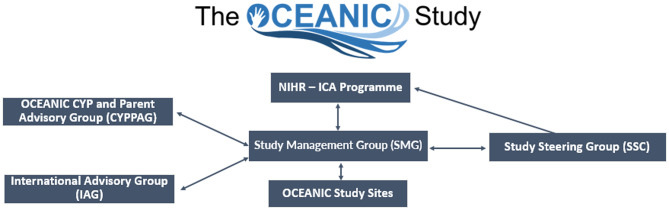
OCEANIC study organogram

The OCEANIC Study CYPPAG were a core part of the governance structure (Fig. 2).

Once data collection had commenced and the group convened, their advice was instrumental in decision making, particularly in helping to overcome recruitment issues. CYPPAG members recommended introducing the study earlier during the PICU admission rather than waiting until families were eligible to consent. As a result, recruitment procedures were modified so that the study was introduced earlier, allowing families additional time to consider participation. Other suggestions included: raising the profile of language services available to staff ensure they could engage with families who do not speak English. They also highlighted that families would be more likely to participate in the research if it were introduced to them by staff they know and trust, so sites were encouraged to utilise this approach, with clinical staff discussing the study with families and then introducing the research delivery staff. Raising the profile of the research through social media was also highlighted as vital.

During CYPPAG meetings, topics of discussion and requests for feedback were numerous. Issues arising, and their impacts, throughout the study were presented to CYPPAG members. Discussions were then facilitated where they were free to share their own relevant experiences and explore how we could learn from/apply aspects of those experiences to the study to enhance the effectiveness of processes. Examples of such topics are provided in Box 1.

**Box 1:** Example CYPPAG suggestions and topics consulted on

- Suggestions for engagement with and retention of participants (variety of methods of data collection, text messaging reminders)
- Study newsletter to be mailed out to participants and content
- Sense checking of qualitative data analysis
- Suggestions for communicating and disseminating the findings
- Involvement in disseminating the study and its findings

**Fig. 3:**
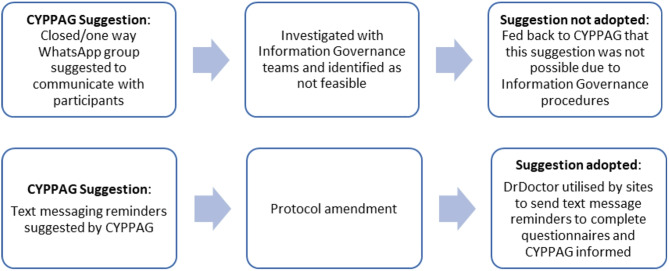
Example CYPPAG suggestions and impact

During the recruitment and data collection phase of the study, meetings had a significant focus on minimising attrition, as this was the most pressing issue at that stage. Members provided numerous suggestions which could help reduce the rate of attrition, including: a closed WhatsApp or Facebook group for the participants; utilising contacts with healthcare providers as a chance to ensure data collection; and the use of text messaging reminders, which the members highlighted aren’t nearly as intrusive as they might be perceived to be. CYPPAG suggestions were carefully considered by the study team and implemented where possible, or when they were not feasible, this was communicated back to the members with an explanation of why (examples shown in Fig. 3).

CYPPAG members also suggested a study newsletter for distribution to participants, as a mechanism for continuing engagement. Newsletters were developed and CYPPAG members commented on their content and wording, making them more engaging and appropriate for a lay audience.

In the data analysis phase of the research, qualitative data were presented to CYPPAG members early in the process of analysis to obtain their feedback on the themes being identified in relation to their own experiences. They were provided with the opportunity to share alternative interpretations and shape the resultant themes.

#### Impact of PPIE

**Fig. 4:**
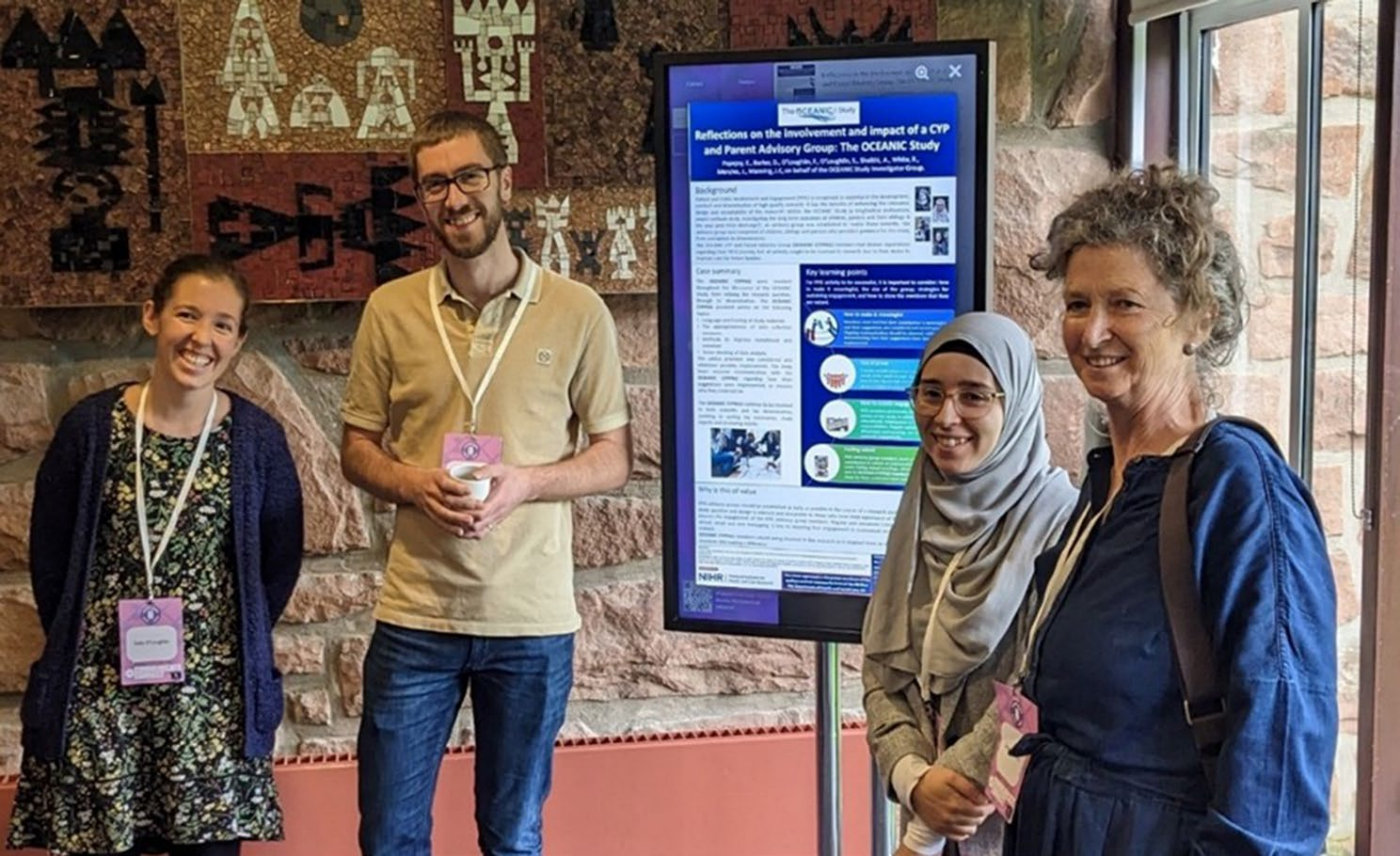
CYPPAG members presenting their involvement in the OCEANIC Study

Since completion of the study, the CYPPAG members have remained engaged with the study, participating in further virtual meetings to plan for dissemination and attended a conference with the study team. This conference attendance both provided them with the opportunity to share their experience of what worked for them in their PPIE experience [22] (Fig. 4) and also hear about the current research being undertaken in the paediatric intensive/critical care setting, a topic which given their own experiences, they are passionate about.

CYPPAG members will remain involved in further dissemination, particularly around the development of lay summaries and sharing events. Some CYPPAG members have also expressed interest in advising on further related research studies and have worked alongside members of the OCEANIC Study team on other projects and as co-applicants for other related funding applications. The establishment of respectful and meaningful relationships from the outset has facilitated these ongoing partnerships which have spanned the best part of a decade and importantly, show no signs of dissolving.

OCEANIC Study CYPPAG members provided positive feedback about their experience of advising on the OCEANIC Study. CYPPAG members felt a sense of pride in their ability to have contributed to research which aims to make a difference to families like their own. They also found that their involvement helped them make meaning of what had been a traumatic experience for them and also felt therapeutic. The impact of their involvement is demonstrated through their own narratives:

*Unexpectedly*,* the meetings were also very therapeutic as it allowed me to talk about my experiences in hospital; the good and bad.* (AS)

*On a personal level*,* being part of this group has helped me to derive a sense of meaning in the ability to be able to use my experiences to help others as well as being able to process my own experiences within the context of the experiences of others. Knowing that the input of the PPIE group has helped to improve the accessibility*,* inclusivity and engagement in the study has been very rewarding. I am extremely proud of being part of this PPIE group and the benefit it has made to this study*,* and I hope that the outputs and follow on working from this will continue to benefit patients and their families as they navigate life post PICU.* (SOL)

CYPPAG members’ experiences demonstrate that when working relationships are built on a foundation of trust, with clear communication channels, they can recognize the tangible difference these make to the study delivery. This helps them to derive a sense of meaning and pride from their involvement. Researchers working in healthcare have a duty to provide these opportunities.

### Reflections and recommendations from the OCEANIC study team

The OCEANIC Study CYPPAG were an integral and valued part of the wider study team. Their involvement exemplified meaningful PPIE, aligning closely with the NIHR’s six standards [4]. Our reflections as a team highlight what contributed to the success of this collaboration and offer recommendations for future research.

1. PPIE was embedded from the earliest stages, prior to funding application, and sustained throughout the study, including dissemination. This long-term integration fostered trust and ensured the relevance of the study. Future studies should plan for PPIE as a longitudinal commitment, not a discrete activity.
2. While time is essential to build relationships, our experience during COVID disruptions highlighted that a shared belief in the research and a collaborative, listening ethos were even more critical. Genuine partnership requires researchers to be responsive, flexible, and open to co-creation. Appropriate costing of PPIE activity ensures that advisory group members feel their contributions are genuinely valued, both in principle and through tangible recognition of their time and expertise. During remote meetings held in the context of the COVID‑19 pandemic, the provision of afternoon tea boxes acted as a shared experience, helping to create a sense of community and connection. This may have contributed to a shared sense of value and appreciation among members.
3. CYPPAG members were oriented to their role and did not require additional support to engage with this role. Learning opportunities were identified and provided to CYPPAG members toward the end of the study, however earlier identification and provision of opportunities could have further enhanced their sense of value and personal impact. We recommend that researchers co-design development and learning opportunities with advisory members from the outset of their involvement, which are tailored to their interests and needs.
4. We found that group size significantly influenced engagement. Groups must be small enough for individual voices to be heard, yet large enough to avoid placing too great a sense of responsibility on a few members.
5. CYPPAG members expressed a desire to be involved in dissemination. Researchers should plan for, and adequately cost into funding applications, advisory group involvement in dissemination activities, including authorship, presentations, and creative outputs, to amplify reach and relevance.
6. Regular assessment of group composition is essential to ensure diversity and inclusion. Whilst some aspects of diversity were achieved within the OCEANIC Study CYPPAG, other areas were less well attended to, including inclusion of CYP, ethnicity, and lower levels of parental education/health literacy. This may have been influenced by the methods of recruitment to the CYPPAG, the small size of the group, and potential age‑related power imbalances within a predominantly adult advisory group. Reflecting on this, explicitly assessing CYP preferences for involvement at the outset and revisiting these throughout the study may help identify more appropriate group compositions, alternative modes of engagement, or tailored approaches to better support CYP participation. To assess and monitor diversity in advisory groups, researchers can use the recently developed Equality Impact Assessment (EqIA) toolkit [ [23]]. An EqIA should be used as a living document, reviewed and updated throughout the study, with periodic recruitment to the group considered to ensure that the range of voices present represents the wider study population. The OCEANIC Study demonstrates that PPIE can be conducted meaningfully, even in a challenging research context.

## Conclusion

The OCEANIC Study highlights the pivotal role of PPIE in paediatric intensive/critical care research. Through the sustained engagement of the CYPPAG, the study demonstrated how meaningful PPIE can enhance research relevance, improve recruitment strategies, and support the dissemination of findings. The active partnership fostered between researchers and PPIE contributors contributed to a deeper understanding of the needs and experiences of children and families following PICU discharge.

This case study highlights the strengths of continuous PPIE through flexible involvement, personal engagement, and proactive communication. It offers a novel model for embedded advisory group collaboration, evidencing how co-production can be maintained across the full research lifecycle. The significance of this work lies in its demonstration of how inclusive and respectful engagement can lead to tangible improvements in study design, data collection, and dissemination, while also providing therapeutic value to contributors. The insights gained from the OCEANIC Study provide valuable lessons for future paediatric research, advocating for PPIE practices that are inclusive, reflective, and impactful. By embedding PPIE into every stage of research, from design to dissemination, paediatric intensive/critical care studies can be more attuned to the real-world needs of patients and families, ultimately contributing to higher quality, patient-centred healthcare outcomes. We hope that through providing this information, we can help other researchers engage more meaningfully with CYP and parent/carers, thus designing and delivering high-quality, impactful research.

## Data Availability

No datasets were generated or analysed during the current study.
